# *De novo* atrial fibrillation post cardiac surgery: the Durban experience

**DOI:** 10.5830/CVJA-2014-067

**Published:** 2014

**Authors:** Dr Ebrahim Mansoor

**Affiliations:** Department of General Surgery, in association with the Department of Cardiothoracic Surgery, University of KwaZulu-Natal, Durban, South Africa

**Keywords:** cardiac surgery, atrial fibrillation, arrhythmia, cardioversion, amiodarone, β-blocker

## Abstract

Atrial fibrillation (AF) is the most common complication post cardiac surgery and results in elevated morbidity and mortality rates and healthcare costs. A pilot, retrospective study of the medical records of all adult patients developing *de novo* AF post surgery was undertaken at the cardiac surgical unit in Durban between 2009 and 2012. We aimed to describe the local experience of AF with a view to suggesting an adapted local treatment policy in relation to previously published data. Fifty-nine patients developed AF during the study period. AF occurred predominantly three or more days post surgery. Thirty-five patients required cardioversion and amiodarone to restore sinus rhythm. Return to the general ward (RGW) was 4.6 days longer than the institutional norm. Liberal peri-operative β-blocker and statin use is currently preferred to a formal prophylaxis strategy. Randomised, controlled trials are required to evaluate measures curbing prolonged length of stay and morbidity burdens imposed by AF on the local resource-constrained environment.

## Abstract

Atrial fibrillation (AF) is the most common arrhythmia after cardiac surgery. This complication constitutes significant morbidity and mortality rates for the cardiac surgical patient.^1^ Consequential increase in length of stay (LOS), partly on the basis of thromboembolic events, incurs a financial burden on health institutions.

Although the entity of de novo AF post cardiac surgery has been intensely studied globally, a grave paucity of data exists from the developing world. The aim of this study was to describe the South African experience of de novo AF post cardiac surgery with special emphasis on the issues pertaining to a resource-limited setting.

## Methods

A retrospective uni-centre audit of a prospectively collated database between December 2009 and February 2012 was undertaken at the Department of Cardiothoracic Surgery, Inkosi Albert Luthuli Central Hospital (IALCH) in Durban, South Africa. All adult patients who developed *de novo* AF post coronary and valve surgery were included in the study. Paediatric patients and patients with chronic pre-operative AF were excluded from the cohort.

Data was extracted from patients’ medical records. Atrial fibrillation was defined as an arrhythmia with irregular irregularity and absent P waves. Diagnosis of AF was confirmed on telemetry and 12-lead electrocardiogram (ECG). AF was managed as per the European Association of Cardiothoracic Surgeons (EACTS) 2006 guideline.^2^ Selective deviations from the guideline were on a patient-specific basis upon consultation with the Department of Cardiology.

The following parameters were evaluated: demographics: age, gender, race; type of surgery: coronary, valve or combinations thereof; risk factors for AF (among others hereunder listed): pre-operative withdrawal of β-blockers, prior cardiac surgery, body mass index (BMI), smoking (within six months prior to surgery); nature of surgery: emergency or elective; co-morbidities: hypertension, diabetes mellitus; time of AF presentation: < 24 hours, 24–48 hours and > 48 hours post surgery; medication: pre-operative use of statins and β-blockers; treatment of AF: none, i.e. spontaneously resolved, electrical cardioversion, amiodarone use, or a combination of cardioversion and amiodarone; pre-operative echocardiographic parameters: left ventricular diastolic dimension (LVD), left atrial size (LA), ejection fraction (EF).

Return to the general ward (RGW) is a surrogate concept introduced to quantify LOS and cost burden imposed by the development of AF in the post-operative period. The institutional norm is two days, one day in the intensive care unit (ICU) and another in the high-care ward, after which time the patient returns to the general cardiac surgical ward.

In addition to the above, follow-up information available at the time of data presentation was analysed, particularly the time from surgery, β-blocker use and maintenance of sinus rhythm were recorded. The study was approved by the Biomedical Research Ethics Committee (BREC) at the University of KwaZulu-Natal (BE296/13).

## Results

Fifty-nine patients developed de novo AF after cardiac surgery in the index cohort during the study period. Considering the total of 997 adult patients who underwent surgical intervention for coronary or valve-related pathology in this period, the institutional AF rate was 5.9%. The number of patients developing AF per surgical procedure is shown in Table 1.

**Table 1:** Incidence of AF per surgical procedure

*Type of surgery*	*Number developing AF*	*Total number of surgeries*	*Percentage of cohort (%)*
Coronary surgery			
Coronary artery bypass graft surgery (CABG)	13	270	45.8
Off-pump coronary bypass surgery (OPCAB)	14	251	
Valve surgery			
Mitral valve replacement (MVR)	7	251	44.1
Aortic valve replacement (AVR)	11	85	
Double valve replacement (DVR)	8	113	
Combination coronary and valve surgery			
CABG + MVR	0	12	10.2
CABG + AVR	6	12	
CABG + DVR	0	3	
Total	59	997	

Thirty-three patients (55.9%) had coronary artery surgery, either alone or in combination with valve surgery. All six patients who underwent combination valve and coronary surgery had aortic valve replacements. Off-pump coronary surgery consisted of 23.7% of the cohort.

Thirty-three patients in the cohort were male and 26 were female. Age ranged from 16 to 82 years (mean: 51.9 years). Thirty-four patients were of Indian descent, while 17 were black and the remaining eight were white patients. The majority (79.4%) of Indian patients had coronary surgery alone or in combination with valve surgery. Fifty-four patients had elective surgery and five had surgery on an emergency basis.

RGW ranged from the institutional norm of two days up to a maximum of 24 days. The mean RGW duration was 6.6 days (*n* = 54). Six patients died during the index admission period, three of whom had emergency surgery.

Twenty-four patients were diabetic and 35 had hypertension. Twenty patients had both diabetes and hypertension.

Thirty-seven patients used β-blockers and 40 were on statin therapy on a chronic basis. Thirty-two patients in the cohort used a combination of statins and β-blockers prior to surgery. β-blockers were withdrawn in only three patients in the immediate pre-operative period.

Miscellaneous risk factors were BMI, previous cardiac surgery and smoking. The BMI ranged from 17 to 42 kg/m^2^, with a mean of 26.4 kg/m^2^ (*n* = 54). Three patients in the cohort had prior cardiac surgery. Twelve patients were still smoking in the immediate pre-operative period. The echocardiographic results are reflected in Table 2.

**Table 2:** Echocardiographic parameters

*Parameter*	*Patient number*	*Range*	*Mean*
LVD (mm)	50	42–75	56.1
LA (mm)	50	33–90	51
EF (%)	52	25–66	52.8

An analysis of the subgroup of patients (*n* = 33) who underwent coronary artery surgery alone or in combination with valve surgery is as follows: mean age was 62.2 years, there were 26 male and seven female, mean BMI (*n* = 31) was 26.9 kg/m^2^, 33 patients were on statins and 31 on β-blockers. Mean echocardiographic parameters (*n* = 27): LVD = 56.3 mm, LA = 45.9 mm, EF = 51.6%.

The coronary patients were a mean of 10.3 years older than the whole cohort and 78.8% were male. The other parameters closely resembled that of the entire cohort.

**Fig. 1.**
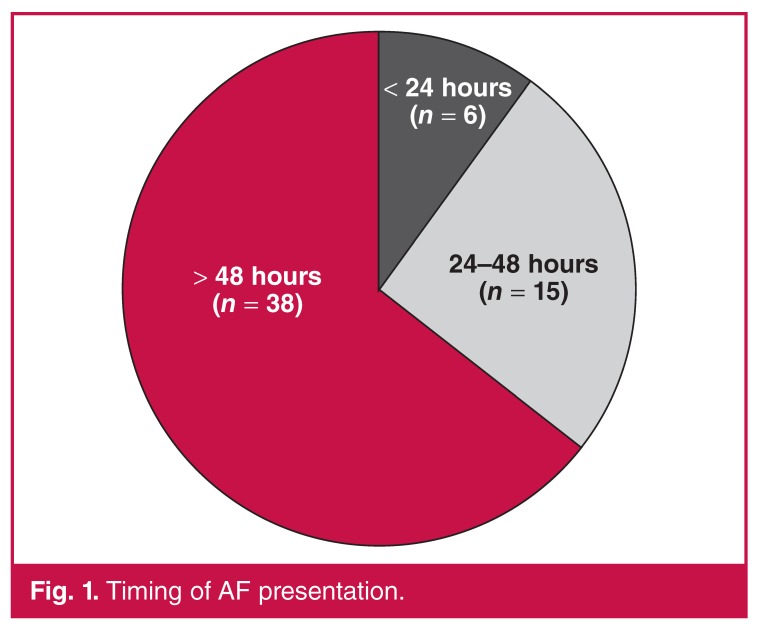
Timing of AF presentation.

**Fig. 2.**
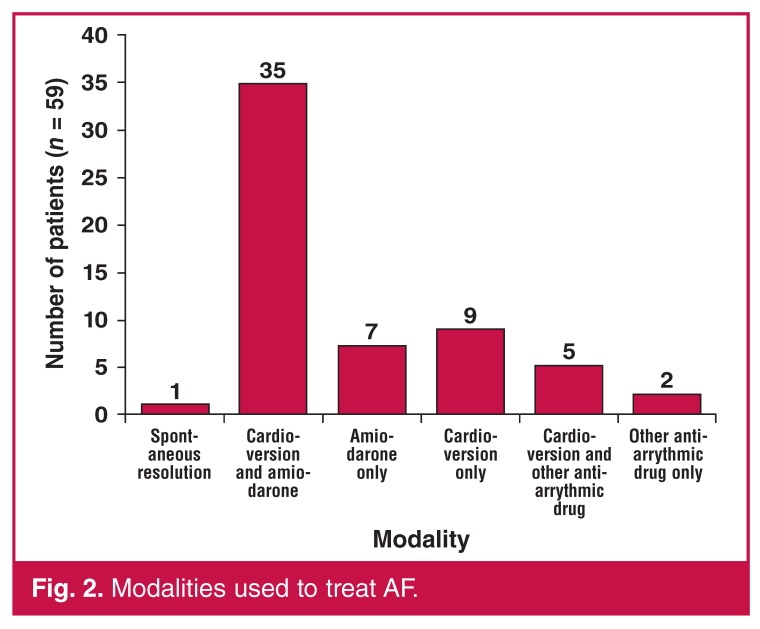
Modalities used to treat AF.

The majority of patients (64.4%) developed AF from day three onwards. The incidence of AF in the individual postoperative time periods is reflected in Fig. 1. The various individual and combination treatment modalities used to treat AF after cardiac surgery is shown in Fig. 2.

Follow-up data was available for 40 patients at the time of data presentation. Follow-up duration ranged from 1.5 to 38 months after surgery, with a mean of 16.1 months. At the follow-up visit, all 40 patients were in sinus rhythm. Twenty-two patients were at that stage noted to be using β-blocker therapy for underlying chronic cardiac conditions.

## Discussion

*De novo* atrial fibrillation post cardiac surgery is a postoperative complication associated with significant morbidity and mortality.^3^ Hakala *et al.* demonstrated, in a retrospective study of 3 676 Finnish patients, an increase in peri-operative cardiovascular accidents (CVA), confusion, ICU LOS and ICU re-admission rates.^4^ Almassi *et al.* showed significantly higher hospital and six-month mortality rates in patients in whom AF occurred.^1^

The mechanisms involved in the development of AF in the cardiac surgical patient are incompletely elucidated. It is probable that pericardial inflammation, catecholamine increase, autonomic disharmony, atrial stretch, metabolic abnormalities, transcellular fluid and electrolyte shifts, and neurohormonal activation act alone or in concert in the production of AF. These factors shorten the atrial refractory period, slowing atrial conduction. Resultant re-entry wavelets bombard the atrioventricular node to produce rapid and irregular ventricular contraction.^5^

Atrial structural alterations, inherent or iatrogenic, affect individual susceptibility to AF. It is widely accepted that the initiation and perpetuation of AF requires a triggering factor and an electrophysiological substrate within the atria. The substrate is mandatory and perhaps explains why some postoperative patients develop AF and some do not.^6^ Furthermore, a genetic predisposition has been proposed in patients with the interleukin-6 promoter gene variant.^3^

AF post cardiac surgery is postulated to arise in a milieu of sympathetic hyperactivity in the post-operative period. Several patient and procedure-related factors have been suggested to confer increased individual vulnerability. The inflammatory state induced by cardiopulmonary bypass, atrial incisions and the relative ischaemia of the atrial septum when cardioplegic solution is delivered via the coronary circulation contribute to the complex processes involved in the generation of AF.^7^

The AF incidence of 5.9% in our cardiac surgical unit is within the widely varied range of incidence reported in most international series, between 5.5 and 65%.^7^,^8^ A meta-analysis of 24 trials estimated the incidence at 26.7%.^9^ A comparison of eight studies with cohorts of 500 or more patients evaluating the incidence and pre-operative risk factors for atrial arrhythmias after cardiac surgery confirmed a comparable incidence between AF post coronary artery bypass surgery (CABG) and post valve surgery, but an increased incidence after combination surgery.^7^

Coronary and valve surgery patients were virtually equally represented in our cohort, however Creswell *et al.* noted that AF incidence was in fact increased in valve and combination valve–coronary surgery.^10^ In the developing world, it is our experience that patients with valve disease present later, once AF has already developed. Since these patients were excluded from our study, this may have accounted for the unremarkable difference in incidence between the valve and coronary surgery groups.

The vast heterogeneity between definitions and study group compositions makes a direct comparison of incidence and other parameters challenging. Several studies do not allude to a definition for AF or method of detection, while others considered only patients requiring intervention for AF. The African incidence of AF post cardiac surgery remains undocumented.

The highest incidence of AF is seen on post-operative days two to three, with fewer patients developing AF in the early post-operative period or beyond four or more days.^7^ Seventy per cent of patients develop AF before the end of day four and 94% before the end of day six.^11^ Mathew *et al.* documented the peak incidence of AF on postoperative days two and three in their prospective, observational study of 4 657 patients undergoing CABG in 70 centres. Fifty-seven per cent of patients had only a single episode of AF during their hospitalisation.^8^

Our study showed that AF was most prevalent from the third post-operative day and not in the immediate post-operative period (< 24 hours). This supports the notion that AF generation is complex and that it extends beyond the peak adrenergic surge prevalent on the first post-operative day.

Tsikouris *et al.* demonstrated P-wave dispersion and atrial conduction time to be greatest on days two to three, and day three, respectively.^12^ It remains unclear whether these electrophysiological alterations contribute wholly or in part to the increased development of AF after the first post-operative day.

A wide array of risk parameters has been evaluated globally, with conflicting results. Mathew *et al.* found age to be an independent risk factor for AF post cardiac surgery and this finding has consistently been reported by others.^7^,^8^ The risk of AF increases by at least 50% per decade and particularly so over 70 years of age. Inflammatory and degenerative changes associated with advanced age cause atrial fibrosis and degeneration.^7^,^13^ The resultant alterations in electrophysiological properties may act as substrates for AF. Most of the parameters considered in this study, albeit few, revealed results that were within normal limits.

Hypertension has been proposed to predict AF after cardiac surgery and this may be related to associated fibrosis and dispersion of atrial refractoriness.^1^,^8^,^11^ However, several well-conducted trials with large numbers of patients have refuted this proposition.^3^,^14^,^15^

Men are more likely to develop AF than women. This disparity may be explained by gender differences in ion-channel expression, and hormonal effects on autonomic tone.^11^,^15^ However, Mathew *et al.* and Echahidi *et al.* expressed a contradictory viewpoint on male predominance.^3^,^8^

Withdrawal of β-blockers pre-operatively in patients on chronic β-blocker therapy causes withdrawal effects, as described by Kalman *et al.*^16^ The poor oral absorption of β-blockers in the post-operative period described by Valtola *et al.* is likely to exarcebate this withdrawal effect.^17^

Beating-heart coronary surgery (OPCAB) did not appear to provide any obvious protective benefit to the development of AF in this study. Notwithstanding the heterogeneity in OPCAB trials, off-pump surgery is still believed to be associated with a significant reduction in post-operative AF.^2^

In our study, only three patients had redo-operations and another three β-blocker withdrawal in the pre-operative period. Therefore no deductions could be made. Twelve patients were noted to be smoking up to the time of surgery. This information was confession-based and furthermore, all patients were advised to cease smoking at the cardiac surgical preview visits.

In their retrospective analysis of 5 058 patients post isolated CABG in patients older than 50 years, obesity was shown by Echahidi *et al.* to be an independent risk factor for AF.^3^ Higher cardiac output requirements, left ventricular mass and left atrial size predisposed to AF in the obese population.^18^ Filardo *et al.* reported a significant relationship between BMI and AF. Their study of 7 027 consecutive patients post CABG with a mean age of 64.9 years showed a median BMI of 28 kg/m^2^.^19^ In our study, a mean BMI of 26.4 kg/m^2^ indicated an overweight population, as per the World Health Organisation, but no relationship was readily apparent between BMI and AF in our cohort.

In our study, all patients were found to be AF free at follow up. The natural history of AF post surgery is that of spontaneous resolution within six weeks, irrespective of treatment modality used. Up to 80% of patients convert to sinus rhythm within 24 hours, even without treatment.^7^ Less than 10% of patients discharged in sinus rhythm develop AF recurrence within six weeks of discharge.^20^ This supports the merit of a more conservative approach locally, especially with haemodynamic stability and low ventricular response. Simple treatment measures such as inotrope reduction, fluid balance management and electrolyte correction (potassium and magnesium) must not be underrated. Anaemia, hypoxia, pain and patient arousal must be addressed as well.

For patients who are haemodynamically unstable, have rapid ventricular responses or features of myocardial ischaemia, initial electrical cardioversion followed by intravenous amiodarone has proved to be a useful strategy locally. We have adopted a rhythm-control strategy as per international trends. A study by Lee *et al.* showed a decreased time to cardioversion, prolonged maintenance of sinus rhythm and decreased overall hospital stay when a rhythm-control strategy was adopted.^21^

In our study, electrical cardioversion was successful only as an isolated modality in nine patients and this is perhaps partially explained by the exclusive use of antero-lateral pad placement.^2^ Direct-current cardioversion is recommended as first-line therapy if AF causes haemodynamic instability or ischaemia. The initial shock energy should start with 300–360 J of monophasic waveform or 200 J of biphasic waveform and results in more than 95% success rate in converting to sinus rhythm.^22^

Amiodarone is the preferred anti-arrythmic agent in our setting as it is readily available, has anti-arrythmic efficiency similar to class I agents, can be used in patients with low ejection fractions, has no pro-arrhythmic tendency and is easily converted to oral medication. Intravenous amiodarone leads to sinus conversion in up to 90% of patients within the first 24 hours.^7^

In our study, amiodarone was administered as an intravenous loading dose (300 mg in 200 ml of 5% dextrose water over 45 minutes, followed by 900 mg in one litre of 5% dextrose water over 24 hours) during the first 24 hours of AF onset after failed electrical cardioversion. This was converted to oral agents (300 mg tds) on post-operative day two (or soonest possible) and weaned off gradually in the subsequent weeks. Rho suggests the continuation of amiodarone for a minimum of one week post surgery since the occurrence of AF beyond day seven is rare.^4^ Amiodarone has proved to be effective in controlling heart rate in the post-operative period and the intravenous preparation is associated with improved haemodynamic status.^23^

Prophylactic amiodarone has inconsistently proved to effect a reduction in post-operative AF. Several trials evaluating the benefit of prophylactic amiodarone included patients concomitantly treated with β-blockers. Mahoney *et al*. showed that intravenous amiodarone is not cost effective for AF prevention if administered to all patients.^15^ Anti-arrhythmic agents other than amiodarone used for the treatment of AF in the study included atenolol, digoxin and diltiazem. These agents are used for rate control in haemodynamically stable patients. The effect of these agents on AF is undoubtedly commensurate with the care with which they are used.

Short-acting β-blockers are the therapy of choice for rate control, especially in ischaemic heart disease, but may be poorly tolerated in asthmatics and patients in cardiac failure.^3^ A meta-analysis of 24 randomised, controlled trials by Andrews *et al*. demonstrated a 77% reduction in AF post CABG.^9^ The protective effect of pre-operative β-blocker therapy is related to the blunting of the high sympathetic tone occurring after cardiac surgery.^16^ Findings of the AFIST II trial suggested that the concomitant use of β-blockers and amiodarone is especially rewarding.^24^ Dunning *et al*. recommended β-blockers for the prevention of AF in all patients undergoing cardiac surgery.^2^ Pre-operative β-blocker withdrawal is a significant risk factor for AF and must be avoided.^5^

Digoxin is grossly inefficient when adrenergic tone is high and is selectively used in patients with reduced ejection fractions.^3^ AV nodal blocking agents such as the non-dihydropyridine calcium channel blockers can be alternatively used for rate control but may cause low cardiac output. These agents must be used cautiously until additional data on their safety profile becomes available.^3^

Historically, several modalities for AF prevention have been used with varying results. These include β-blockers, amiodarone, digoxin, bi-atrial pacing, calcium channel blockers, magnesium, statins, N-3 polyunsaturated fatty acids (PUFAs) and antiinflammatory agents.^3^

We do not use any AF prophylaxis strategy in our unit. There are no robust risk models or evidence available to govern such a strategy. Moreover, prophylaxis has not been clearly shown to positively impact on morbidity or mortality arising from AF, and we are unaware of any feasibility studies supporting a prophylaxis strategy in resource-constrained environments.^3^ The optimal anti-arrhythmic agent, dose, timing of initiation, and route and duration of drug administration for prophylaxis remain elusive.

β-blockers are used liberally for our cardiac surgical patients, mainly for coronary artery disease, as evidenced in this cohort, barring contra-indications. We are currently content to continue β-blocker use in the peri-operative period and enjoy whatever consequential AF reduction it may confer.

For the treating surgeon in the developing world, AF is a harbinger of increased LOS and resultant cost burdens.^4^,^11^,^14^ The patients in our cohort took 4.6 days more than the institutional norm to return to the general ward. The cost implication is likely to be significant in light of the strain imposed on intensive-care and high-care wards.

In a study in the United States of America conducted by Aranki *et al.*, LOS was increased by 4.9 days, with additional hospital costs amounting $10 000–11 500.^11^ AF results in longer ICU and overall hospital stays, even after adjusting for severity of illness.^7^ Tamis *et al.* showed an increase in LOS of 3.2 days independent of variables.^25^ It is, however, possible that the RGW status in our cohort was affected by other disease processes unrelated to AF.

Statins have been observed to attenuate inflammation and reduce AF post coronary surgery.^26^ The ARMYDA-3 trial was a prospective, randomised study that showed atorvastatin 40 mg daily, commenced seven days prior to elective surgery on cardiopulmonary bypass and continued in the post-operative period, reduced the incidence of AF by 61%.^27^

An exciting aspect of de novo AF post cardiac surgery not addressed by this study requires discussion; the role of cardiac biomarkers and risk-prediction models in the prediction of post-operative AF. Studies by Gasparovic *et al.* and Pilatis *et al.* showed elevated B-type natriuretic peptide (BNP) levels to be predictive of AF in patients undergoing CABG.^28^,^29^ The utility of biomarkers in the setting of AF post cardiac surgery requires further clarification prior to a recommendation on their use but preliminary studies certainly show promise for AF prediction and thromboembolic risk stratification.

With regard to risk-prediction models for the development of AF, several have been developed and incorporate risk factors, some of which are mentioned in Table 3. These models have thus far provided controversial and inconsistent results, which have limited their widespread adoption. However, studies by Chua *et al.* and Baker *et al.* demonstrated the CHADS_2_ and CHA2DS_2_-VASc scoring systems to be predictive of AF post cardiac surgery.^30^,^31^ The limitations of these studies are that they were retrospective in nature, the sample size was small, and the patient population was heterogeneous. A recent study by Sareh *et al.* showed the CHADS_2_ score to be a powerful and convenient predictor of post-operative AF in a cohort of 2 120 patients.^32^

A large, prospective, multicentric trial will provide a definite answer as to whether the CHADS_2_ and the CHA_2_DS_2_-VASc scoring systems reliably predict post-operative AF. Should this be proved to be so, physicians will be guided to develop an effective prophylaxis strategy, including drugs and perhaps even prophylactic ligation of the left atrial appendage for ‘high-risk’ patients.

Limitations of this study are: it described only post-operative patients developing AF and an analysis of a control group was not undertaken. Furthermore, the cohort number was small relative to other similar studies conducted internationally.

## Conclusions

**Fig. 3.**
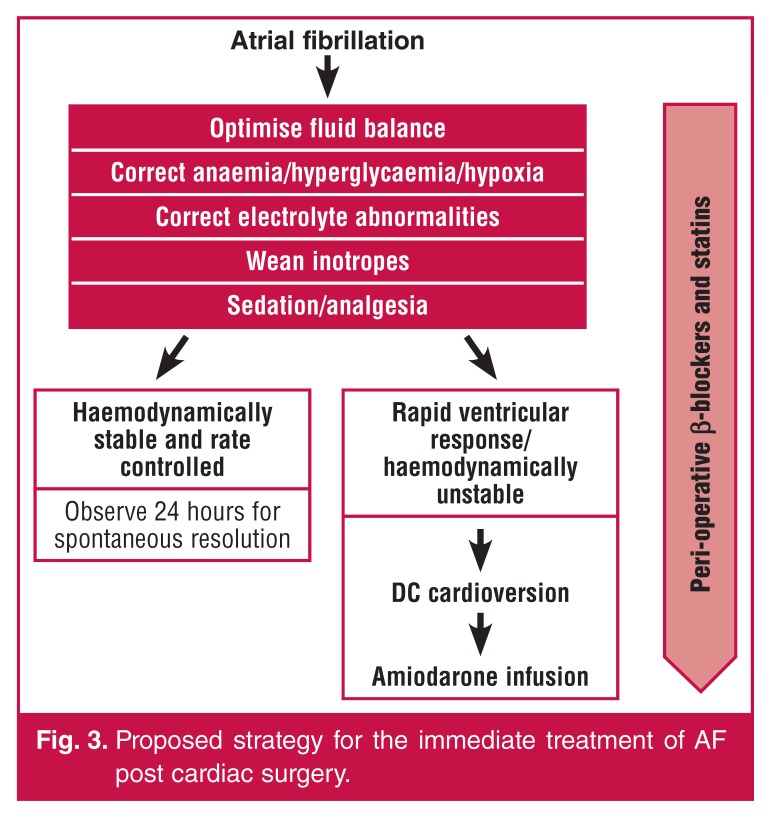
Proposed strategy for the immediate treatment of AF post cardiac surgery.

This study serves to add to the growing body of information regarding *de novo* AF post cardiac surgery and provides some insight into the problem in developing countries. We propose a simple algorithm, shown in Fig. 3, for the immediate postoperative treatment of AF. Experience locally appears to mirror that of international cardiac surgical units. The aetiopathogenesis of AF is complex and a plethora of risk factors have been proposed (Table 3).^33^

**Table 3:** Risk factors for AF

Pre-operative
Advanced age
Male gender
Hypertension
Previous AF
History of previous cardiac surgery
Congestive heart failure (CHF)
Chronic obstructive pulmonary disease (COPD)
Right coronary artery (RCA) disease
Peripheral vascular disease
Left ventricular hypertrophy (LVH)
Left atrial enlargement
Electrocardiographic features
Renal failure
Moderate or severe aortic atherosclerosis
Withdrawal of β-blocker or ACEI
Body surface area (BSA)
Obesity and metabolic syndrome
Intra-operative
Aortic cross-clamp time
Bicaval canulation
Pulmonary vein venting
Type of surgery
Need of perioperative IABP
CPB time
CPB inclusive of cardioplegic arrest
Systemic hypothermia
Post-operative
Respiratory compromise
Red cell transfusion

Use of the CHADS2 and CHA_2_DS_2_-VASc scoring systems and cardiac biomarkers as AF predictors appear promising. Liberal peri-operative β-blocker and statin administration is currently highly recommended. AF prophylaxis for the elderly, obese Indian male undergoing coronary surgery locally requires validation. Opportunistic surveillance for AF is advised at follow-up cardiology visits. Well-designed prospective studies are required for the better understanding and treatment of this common post-operative complication locally. The developing world should concentrate study efforts on LOS and cost reduction.
